# Influence of the indirect restoration design on the fracture resistance: a finite element study

**DOI:** 10.1186/s12938-015-0115-4

**Published:** 2016-01-08

**Authors:** May Lei Mei, Ya Ming Chen, Hao Li, Chun Hung Chu

**Affiliations:** 1 Faculty of Dentistry, The University of Hong Kong, 34 Hospital Road, Hong Kong SAR, China; 2 Institute of Stomatology, Nanjing Medical University, Nanjing, China; 3 School of Civil and Hydraulic Engineering, Hefei University of Technology, Hefei, China

## Abstract

**Objectives:**

To establish a three-dimensional (3D) finite element (FE) model of a maxillary first premolar and to evaluate the stress generated on the tooth (dentine) and on the indirect composite resin restorations by occlusal forces.

**Methods:**

An embedded intact maxillary first premolar tooth was sliced serially and scanned digitally parallel to the occlusal surface. The 64 images were assembled in a 3D FE mesh and exported to generate a 3D solid tooth model. Simulated, adhesively cemented indirect mesial-occlusal-distal (MOD) inlays of 2 mm (I1), 3 mm (I2) and 4 mm (I3) in width, and MOD onlays with occlusal cusp coverage of 2 mm (O1) and 3 mm (O2) in depth were created. The peak von Mises stress values in the five tooth models resulting from static vertical and oblique occlusal forces (300 N) were evaluated using Patran FE software.

**Results:**

The peak stress values generated by vertical occlusal force generated in dentine of I1, I2, I3, O1 and O2 restoration were 67, 32, 29, 38 and 27 MPa, respectively, and those generated by oblique occlusal force were 52, 114, 168, 54 and 55 MPa, respectively. The peak von Mises stress values in I1, I2, I3, O1 and O2 restoration subjected to oblique occlusal loading were 79, 120, 1740, 1400 and 1170 MPa, respectively.

**Conclusion:**

A 3D FE model of a maxillary first premolar was established. Simulated cemented composite resin onlay markedly reduces occlusal stress in the underlying dentine of large MOD preparation. Oblique occlusal force imparts substantially higher stress to large composite resin inlay than to the adjacent dentine.

## Background

An increased interest in aesthetic dentistry and concerns about mercury in amalgam alloys have led to an increased demand from patients for metal-free restorations and a relatively simple way to restore their teeth [1]. Though the use of posterior direct composite resins is still the common practice, such restorations may lead to problems such as fracture and microleakage caused by polymerization shrinkage [2]. Those properties of direct resin may lead to postoperative sensitivity, marginal staining and secondary caries [3, 4]. Following secondary curing, indirect composite resin inlays and onlays generally show improvements in their degree of polymer conversion, mechanical properties, wear resistance and marginal microleakage [5, 6]. Correct tooth morphology also may be achieved with less difficulty for large indirect restorations.

However, the use of indirect inlays to replace failed mesial-occlusal-distal (MOD) direct placement restorations in premolar teeth is questionable on biomechanical grounds [6, 7]. Concerns arise from the fact that removing and replacing such defective intracoronal restorations can lead to a significant extension of the original preparations, with an increased size, isthmus width and cusp height [8, 9]. Occlusal forces on an inlay produce stresses along the sides and base of the restoration, which may fracture the tooth [10]. Covering the occlusal surface of the intracoronal MOD preparation with metal to form an onlay is found to greatly reduce the potentially damaging effects of the occlusal forces [11]. Tooth fractures occur less readily in intact posterior teeth than following cavity preparation [12, 13]. The width and depth of premolar preparations are important factors affecting tooth strength [14, 15]. The type of cavity preparation and materials also affect the extent of tooth fracture [16, 17].

Early finite element (FE) studies found that increased cavity dimensions were associated with increased stresses in the remaining tooth structure [18–20]. More recent FE studies have evaluated several parameters of occlusal forces, MOD cavity preparation geometry and dental materials used to restore maxillary premolar teeth [20, 21]. Teeth restored with ceramic materials have showed a higher fracture resistance than those restored with composite resin in several FE studies [22, 23]. On the other hand, composite resin restorations like inlay and onlay also have their own fields of application. They are less costly, time-saving, and easy to remove when subsequent restorative treatment is necessary. Since composite resin has a lower fracture resistance than ceramics [22, 23], the optimization of cavity dimension becomes critical in making a successful resin restoration. Therefore, the question of how to control the dimension of the resin restoration was raised in the current study. Few FE studies have investigated the effects of different dimensions of resin inlay and onlay on risk of tooth fracture under different loading forces.

The objective of this study was to establish a 3D FE model of a human maxillary first premolar tooth and to use this model to evaluate the effects of (a) MOD inlay restorations of different occlusal isthmus widths and (b) MOD onlay restorations of different occlusal cusp coverage depths on the stresses generated within the tooth model and restorations from occlusal forces. The null hypothesis proposed is that, when subjected to either a vertical or oblique standardized static occlusal force, there are no marked differences present in the resulting peak von Mises stress values for different sizes of indirectly fabricated MOD composite resin inlays and onlays restoring a maxillary premolar tooth model.

## Methods

An intact human maxillary first premolar tooth extracted for orthodontic reasons was selected as the generic model. The restored tooth was at risk for cuspal fracture in clinical practice [24]. The crown dimensions of the selected tooth were similar to average values [25].

The tooth was embedded in clear acrylic resin (ProBase^®^Cold, Ivoclar vivadent, PR Liechtenstein), then sliced serially at 0.3 mm increments along its long axis from cusp tip to root apex using a thin diamond saw (Isomet^®^Low Speed Saw, Buehler Ltd, IL, USA) with copious water cooling. Each of the 64 sections obtained was scanned from the occlusal side using a digital flatbed scanner (S2W4300/3300u, BenQ Asia Pacific Corp., Taipei, Taiwan), and the images were stored as BMP files with a resolution of 1200 dots per inch (dpi). The images were assembled in a 3D wire-frame mesh structure by means of a computer software program (Photoshop 7.0, Adobe Systems Inc., San Jose, CA, USA). The 3D curves were exported using proprietary software (Rhino™ 3D, McNeel North America, Seattle, WA, USA) to generate a maxillary first premolar solid tooth model (enamel, dentine, pulp chamber and periodontal ligament) by fitting the horizontal and vertical profiles. The solid model of the maxillary premolar was subsequently exported as XT files (Patran™ 2004, MSC Software Corp, Santa Ana, CA, USA) to establish the 3D FE model. Following proportional adjustments, a virtual reality image with a criss-crossing mesh was established.

**Fig. 1:**
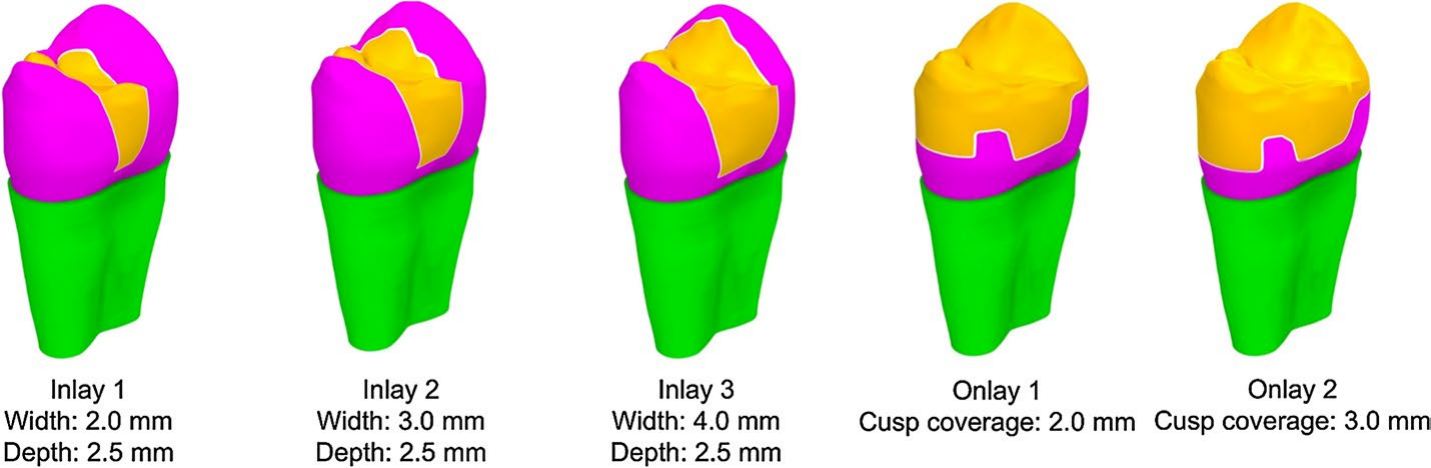
3D models of the inlay and onlay restoration designs

**Fig. 2:**
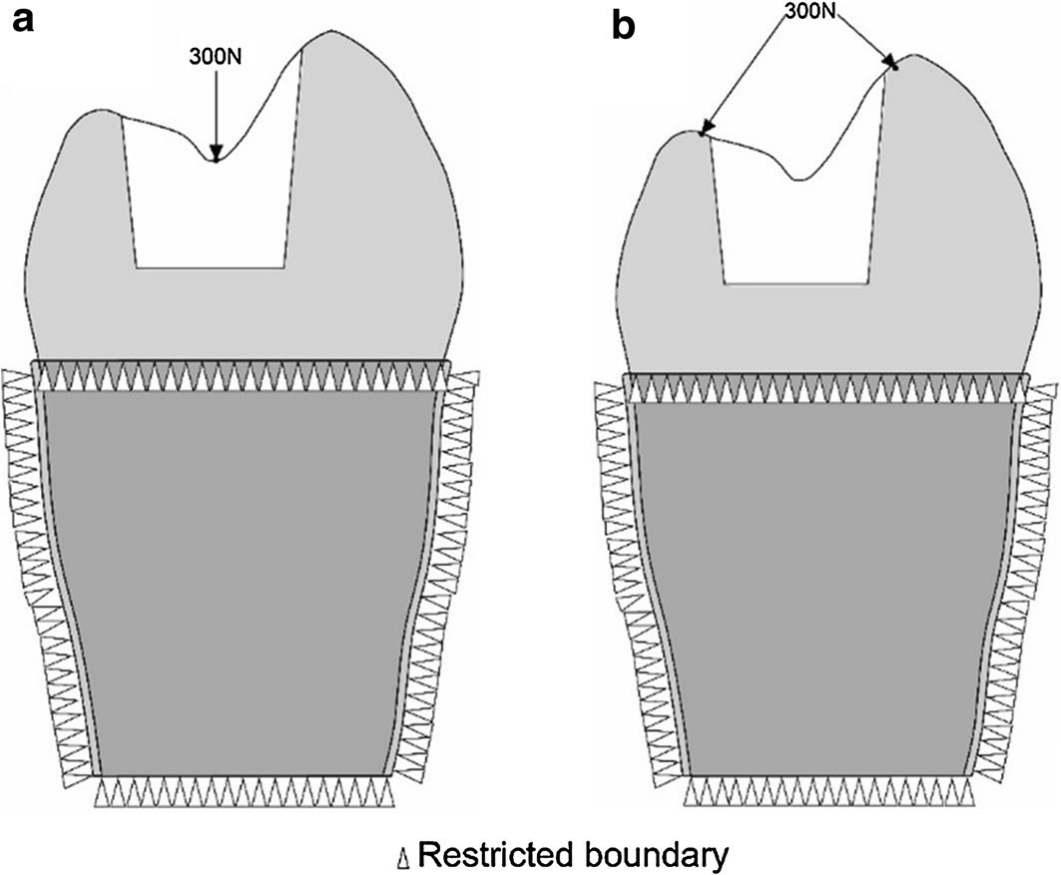
Illustration of load and boundary conditions: **a** vertical loading; **b** oblique loading

The 3D solid model was used to create simulated MOD inlay preparations of three different occlusal widths: I1 (2.0 mm), I2 (3.0 mm), I3 (4.0 mm). The occlusal depth of each cavity preparation was 2.5 mm, and the gingival margins of the mesial and distal proximal boxes were placed 1.0 mm above the enamel-cemental junction. The cavity floors were flat, and the cavity walls had a 5° divergence angle. No cavosurface bevels were present (Fig. 1). The 3D solid model was also used to create simulated cusp coverage MOD onlay preparations of two different occlusal cusp coverage depths: O1 (2.0 mm) and O2 (3.0 mm) (Fig. 1). The original dimensions of each cavity preparation before cusp reduction were as for I3. An 80-µm-thick resin-based adhesive cement layer was established between the cavity walls and simulated indirectly fabricated proximal (Class II) composite resin restorations. A 130-µm-thick periodontal ligament was also established [26]. The solid models were imported into Patran 2004, redefined and meshed using a 10-noded tetrahedral element, resulting in 55,032 nodes and 37,010 elements. Sufficiently fine meshing was taken to ensure the mesh convergence of the results. Different material properties were assigned to the elements (Table 1) according to their volume definitions. All materials were assumed to be homogeneous and isotropic. Five different models were generated. To study the effects of different cavity designs, only one material of the restoration was defined to suppress interference from other factors. The boundary conditions were specified to be consistent with physiological conditions. They were set as fixed, vertical and horizontal displacements along root surfaces of the model that were restricted to simulate support from the alveolar bone [21] (Fig. 2).

**Table 1:** Properties attributed to the tooth structure and restorative material

Material	Young’s modulus (GPa)	Poisson’s ratio
Enamel^1^	48	0.30
Dentine^1^	18	0.31
Periodontium^1^	0.07	0.40
Composite resin^2,a^	14	0.30
Resin cement^2,b^	6	0.30

^1^Ausiello et al. [37]; Couegnat et al. [21]

^2^Bisco, Inc., Schaumberg, IL, USA (^a ^Tescera, ^b ^C&B Cement)

Two types of static loading were used: (a) vertical loading on the occlusal surface perpendicular to the loading axis (Fig. 2a, b) oblique loading resolved into two oblique components acting perpendicularly to the inner cusp inclines, which lay at approximately 45° to the vertical loading axis (Fig. 2b). The force applied for both types of loading was 300 N [27]. The von Mises stress was used as the study variable, based on the distortion energy theory in engineering, using the formula $\sigma_{v} = \sqrt[{}]{{\frac{{\left( {\sigma_{1} - \sigma_{2} } \right)^{2} + \left( {\sigma_{2} - \sigma_{3} } \right)^{2} + \left( {\sigma_{3} - \sigma_{1} } \right)^{2} }}{2}}}$ [28]. The equivalent stress $\sigma_{v}$ is referred to as Von Mises stress. The variables $\sigma_{1}$, $\sigma_{2}$ and $\sigma_{3}$ are referred to as the three principal stresses. Whatever the state of stress, when the ratio of the deformation reaches a critical value related to the property of the material, then the material begins to yield.

The peak von Mises stress was evaluated under two loading conditions (oblique and vertical) for each of five restorations (3 inlays and 2 onlays). Subsequently, the peak von Mises stress in term of oblique loading condition was analysed on dentine and restorations, respectively. In addition, the von Mises stress patterns under two loading conditions (vertical and oblique) were also assessed.

## Results

**Fig. 3:**
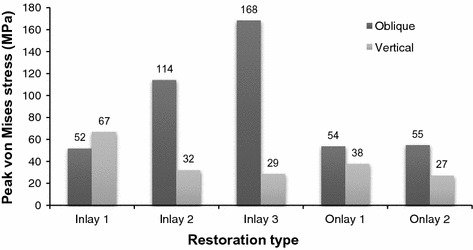
Peak von Mises stress values in the dentine for the five models subjected to vertical and oblique occlusal loadings of 300 N

Figure 3 shows the peak von Misses stress value of dentine for the five models subjected to vertical and oblique occlusal loading. I1 has a slightly lower stress value (52 MPa) under oblique loading than under vertical loading (67 MPa). I2 has an obviously higher stress value under oblique loading (114 MPa) than under vertical loading (32 MPa). I3 has a similar pattern to I2. Similar patterns were also observed in both O1 and O2; namely, stress value was slightly higher under oblique loading than under vertical loading. In general, Peak von Mises stress values in the dentine increased for the oblique force but decreased for the vertical force, with increasing inlay cavity width in particular and with increasing onlay cusp coverage depth. Except for the 1.0-mm-wide I1, all of the other models demonstrated higher stress values resulting from oblique loading than from vertical loading. When subjected to an oblique force, peak von Mises stress values in the inlay restorations increased markedly for the 4.0-mm-wide I3 (168 MPa), being 3.25 times that for I1 (52 MPa) and 1.47 times that for I2 (114 MPa). Peak stress values also were high in the two onlay restorations, though they were lower for O2, which had an increased cusp coverage depth of 3.0 mm.

**Fig. 4:**
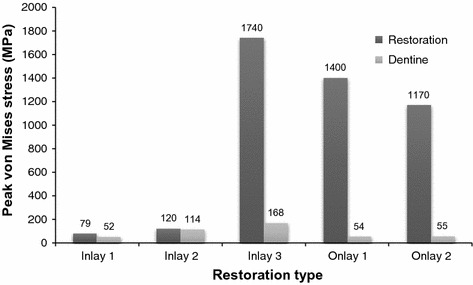
Peak von Mises stress values in the dentine and restorations for the five models subjected to oblique occlusal loadings of 300 N

For the five models, when subjected to an oblique force, the peak von Mises stress values of the restoration and dentine are shown in Fig. 4. I1 and I2 show much less stress, not only on the restoration but also on the dentine, whereas the very wide I3 and the two onlays demonstrated much higher stress values in the restorations than in the dentine.

**Fig. 5:**
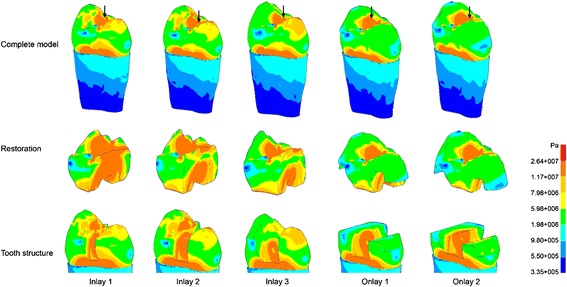
Von Mises stress patterns for the five models subjected to vertical occlusal loadings of 300 N; *arrows* indicate the locations of peak von Mises

**Fig. 6:**
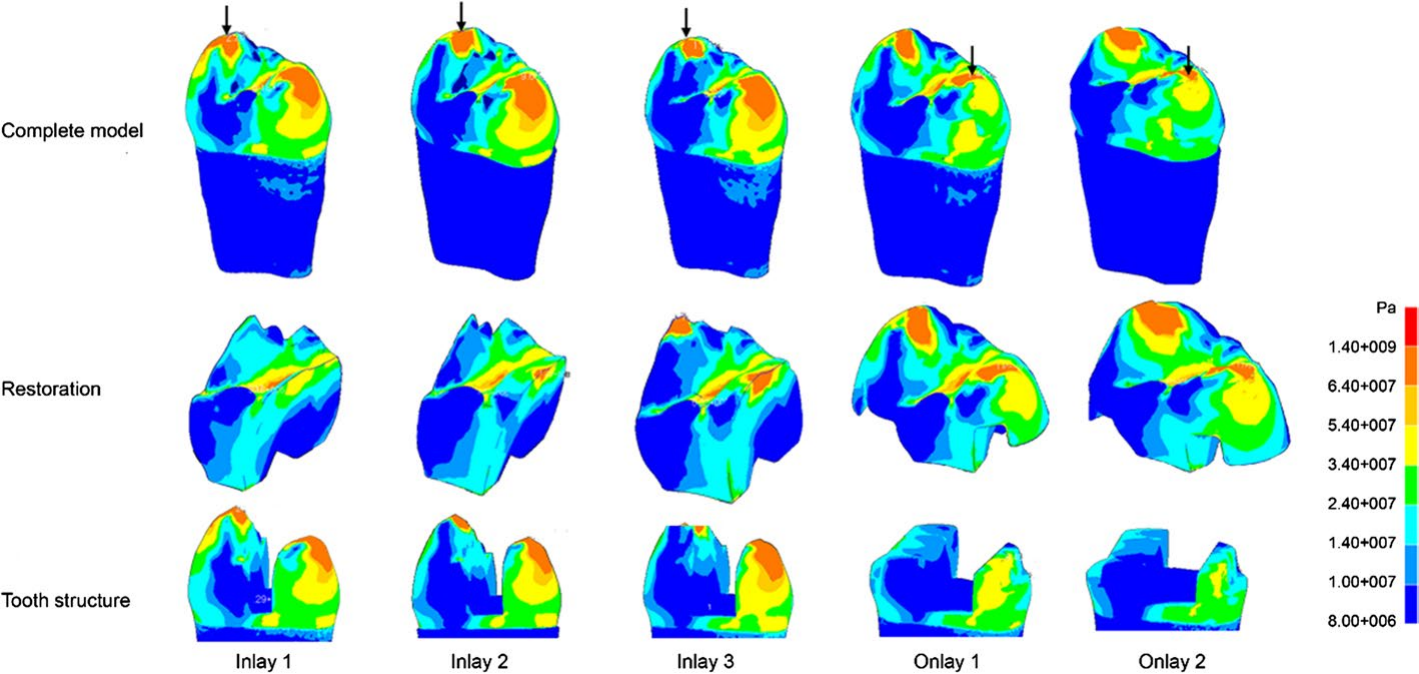
Von Mises stress patterns for the five models subjected to oblique occlusal loadings of 300 N; *arrows* indicate the locations of peak von Mises

The distribution of the von Mises stress patterns in the five models for the vertical and oblique forces (300 N) are illustrated in Figs. 5 and 6, respectively. The stress distributions of the inlay groups are similar, even with the vertical occlusal loadings and high stress located on both buccal and lingual cusps. In the onlay groups, however, the stress on the cusps has been remarkably reduced. This may reduce the risk of tooth fracture. Moreover, the enamel-dentine junction and bottom of the cavity are also the area with high stress under vertical force. For an oblique force, the highest stresses in the cuspal dentine were associated with the widest inlay preparation.

## Discussion

Indirect restorations are those that fit within the anatomic contours of the clinical crown of a tooth. They can be classified as inlays or onlays, depending on whether they have an occlusal veneer or not [29]. The biomechanical study of a tooth with an indirect restoration presents difficulties in many respects, such as the complexity of the tooth’s structure, restorative materials, boundary condition, cavity shape, dimensions and state of stress [20]. This study investigated the stress distribution of indirect restorations of different designs by means of an FE method. The reality was only reproduced by a mathematical approach using typical material properties, loading and boundary conditions.

To keep the simplification effect as low as possible, calculations were not limited to the tooth and the restorations but extended to other structures such as the periodontal ligament and the alveolar bone. Though computerized tomography techniques are available for anatomical and structural data collection [30, 31], obtaining digital images of the premolar tooth in the present study was far simpler. Conventional computer-aided-design (CAD) software is able to represent the curvature of the real object [18, 32]. One of the concerns of the study is that the fracture is due to a static loading, while fatigue phenomena should be considered. In the current study, the conditions and testing of the stress distribution of the materials and tooth have been simplified to instant force. Therefore, the results cannot be extrapolated to the in vivo situation, and caution should be exercised in their interpretation.

Based on the results of this study, the null hypothesis was not accepted. In the present study, as the size of the cavity and the inlay increased, the stress caused by oblique occlusal loading on the dentine increased more dramatically than that caused by vertical occlusal loading. This indicated that oblique occlusal forces in particular imparted high stresses to adhesively cemented large MOD composite resin inlays and onlays and, to a much lesser extent, to the functional remaining cusp structures present with the large inlay restoration (~160 MPa). Moreover, when a tooth with an onlay was subjected to oblique forces, the stresses could be effectively transferred to the composite resin onlay restorations rather than to the underlying tooth structure. Under oblique occlusal loading, the peak von Mises stress values in the dentine increased with the width of the inlay cavity width from I1 to I3. When the inlay was replaced by an onlay with the same cavity width, the peak von Mises stress on dentine decreased. Moreover, in large inlays and onlays under oblique occlusal force, the stress in restorations was substantially higher than the stress in dentine. This suggested that the onlay restorations offer protection to the underlying tooth structure. The Young’s modulus of the composite resin used is 14 GPa, which is similar to that of dentine (18 GPa) and less than that of porcelain (70 GPa) or Ni–Cr alloy (204 GPa) [33]. It was also shown that a less rigid restoration could relax the applied stress by means of greater elastic deformation [34]. The less rigid composite resin shows that a greater elastic deformation would result in a lower deformation of the cusps [34]. Therefore, the risk of tooth fracture associated with large cavity preparations could be reduced by the overlaying of the vulnerable cusps with proper restorative materials [35].

Different load conditions result in different concentrations of stress on a tooth. Oblique occlusal force induces a high stress concentration on the cusps and increases the risk of tooth fracture (Fig. 6). In inlay groups, the stress in dentine under oblique force was higher than that under vertical force, and the difference increased with the width of the inlay. The tooth cusps become narrow when the width of the inlay is large [22, 36]. The stress on dentine reduced remarkably when the cavity was restored by O1, which had the same width as I3 (Fig. 3). The high stress concentration on the cusps when they were restored by inlays was also relieved in onlays. Therefore, this study confirmed that preparing the cusps and making an onlay restoration for a large cavity could reduce the high stress generated by occlusal loading. In addition, the difference of stress values and distributions was not significant in the two onlay groups, therefore O1, which had a small depth, was preferred because it was more conservative than O2, which had a large depth.

## Conclusions

In this study, a 3D FE model of a maxillary first premolar tooth was established for evaluation of the stresses generated within the tooth and the indirect restoration from occlusal forces. Oblique occlusal force imparted substantially higher stress to large composite resin inlay than to the adjacent dentine. Onlay preparation reduced occlusal stress and the risk of cusp fracture associated with large cavity preparation. Therefore, an onlay with cusp coverage should be used for restoration when the width of the cavity reaches 4 mm.

## Abbreviations

MOD: mesial occlusal distal
FE: finite element
CAD: computer-aided design
